# Use of Dual RNA-seq for Systems Biology Analysis of *Zea mays* and *Aspergillus flavus* Interaction

**DOI:** 10.3389/fmicb.2020.00853

**Published:** 2020-06-03

**Authors:** Bryan Musungu, Deepak Bhatnagar, Sylvie Quiniou, Robert L. Brown, Gary A. Payne, Greg O’Brian, Ahmad M. Fakhoury, Matt Geisler

**Affiliations:** ^1^Department of Plant Biology, Southern Illinois University, Carbondale, IL, United States; ^2^Southern Regional Research Center, USDA-ARS, New Orleans, LA, United States; ^3^Warm Water Aquaculture Research Unit, USDA-ARS, Stoneville, MS, United States; ^4^Department of Plant Pathology, North Carolina State University, Raleigh, NC, United States; ^5^Department of Plant Soil and Agriculture Systems, Southern Illinois University, Carbondale, IL, United States

**Keywords:** interactome, maize, *Aspergillus flavus*, aflatoxin, gene regulatory network

## Abstract

The interaction between *Aspergillus flavus* and *Zea mays* is complex, and the identification of plant genes and pathways conferring resistance to the fungus has been challenging. Therefore, the authors undertook a systems biology approach involving dual RNA-seq to determine the simultaneous response from the host and the pathogen. What was dramatically highlighted in the analysis is the uniformity in the development patterns of gene expression of the host and the pathogen during infection. This led to the development of a “stage of infection index” that was subsequently used to categorize the samples before down-stream system biology analysis. Additionally, we were able to ascertain that key maize genes in pathways such as the jasmonate, ethylene and ROS pathways, were up-regulated in the study. The stage of infection index used for the transcriptomic analysis revealed that *A. flavus* produces a relatively limited number of transcripts during the early stages (0 to 12 h) of infection. At later stages, in *A. flavus*, transcripts and pathways involved in endosomal transport, aflatoxin production, and carbohydrate metabolism were up-regulated. Multiple WRKY genes targeting the activation of the resistance pathways (i.e., jasmonate, phenylpropanoid, and ethylene) were detected using causal inference analysis. This analysis also revealed, for the first time, the activation of *Z. mays* resistance genes influencing the expression of specific *A. flavus* genes. Our results show that *A. flavus* seems to be reacting to a hostile environment resulting from the activation of resistance pathways in *Z. mays*. This study revealed the dynamic nature of the interaction between the two organisms.

## Introduction

*Zea mays* is one of the three largest cereal crops in the world (*Z. mays*, *O. sativa*, and *T. aestivum*) in terms of annual production and in the United States alone 13.8 billion bushels of corn was produced for the year 2020. It is also crucial as a staple crop that feeds millions of people and animals daily. However, corn yields are affected by diseases caused by pests, including fungal pathogens such as *Aspergillus flavus*. *A. flavus* is an opportunistic pathogen that adopts a necrotrophic lifestyle, causing cell death in the host and feeding on dead host tissue. *A. flavus* has also been shown to infect multiple crops such as *Gossypium hirsutum*, *Arachis hypogaea*, and *Prunus dulcis* (Bedre et al., 2015; Fountain et al., 2015a). In addition to its effect on plant health, *A. flavus* can also affect animal and human health due to the production of aflatoxins in infected crops (Nesbitt et al., 1962; Cole, 1986, Ostry et al., 2017). Aflatoxins, notably aflatoxin B1, are potent mycotoxins and potential carcinogens when consumed by animals. Hence, many countries have adopted laws that restricting the marketing of crops that are contaminated with aflatoxins in certain national or international markets. Additionally, an intensive effort has been made to study the *A. flavus* - *Z. mays* interaction at multiple levels to identify factors involved in crop yield loss. Similarly, extensive research has been conducted to study the life cycle and biology of the pathogen *A. flavus* itself in genes such as VeA and LaeA (Amare and Keller, 2014; Fountain et al., 2015a, Tang et al., 2015).

The results from genome comparison with other *Aspergillus* species have led to the identification of several genes that are keys to the production of secondary metabolites and other pathogenicity factors in *A. flavus*, (Rokas et al., 2007; Ehrlich and Mack, 2014). The *A. flavus* aflatoxin gene cluster has been elucidated by mutational studies. Some genes in this cluster have also been linked to multiple developmental processes (Yu et al., 2004; Price et al., 2006). At the cellular level, the velvet complex is made up of multiple developmental genes that also have an effect on secondary metabolism (Calvo et al., 2004). Likewise, *LaeA*, another developmental gene in *A. flavus*, linked to pathogenesis and secondary metabolism, was identified through comparison to *A. nidulans* (Kale et al., 2008; Amaike and Keller, 2009, Chang et al., 2013). Other studies have highlighted the importance of hydrolytic processes and cellular transport at the cellular level during pathogenesis, including the development of aflatoxisomes, special organelles that harbor enzymes essential for the biosynthesis of aflatoxins in *A. flavus* (Kistler and Broz, 2015). Early work attempting to understand the crosstalk between *A. flavus* and *Z. mays* led to the identification of lipoxygenases in both *A. flavus* and *Z. mays* as key genes in this process (Brown et al., 2009). Since then, the girth of *Aspergillus* genomic data has allowed the discovery of many new interactions highlighting the involvement of proteins such as the *A. flavus* small ubiquitin-like modifiers *PdeH* in the interaction between *A. flavus* and *Z. mays* (Nie et al., 2016; Yang et al., 2017).

Genomic studies with *Z. mays* have also been successful in elucidating mechanisms controlling resistance to fungal pathogens. Unlike *A. flavus*, there are multiple post-genomics resources available to *Z. mays* researchers, such as publicly available interactomes, that can be used to associate complexes and pathways with interacting proteins (Musungu et al., 2015). Nevertheless, recent studies investigating resistance of *Z. mays* to *A. flavus* almost exclusively involved utilizing singular genomics approaches (using information from one genome at a time) such as RNA-seq and DNA-seq. These have been able to identify and analyze key maize disease resistance proteins such as PR10, PR5, chitinases, trypsin inhibitors, and a vast array of other genes that contribute to the resistance in *Z. mays* to *A. flavus* and other pathogens (Brown et al., 1995; Chen et al., 2007). Moreover, recent “single genomics” transcriptomic studies have shown that the activation of key pathways, such as the jasmonate, ethylene biosynthesis and several other signaling pathways, are implicated in the resistance of *Z. mays* to *A. flavus* (Burow et al., 1997; Scarpari et al., 2014, Christensen et al., 2015). Breeding and genetic marker analysis efforts using genome wide association and quantitative trait loci studies indicate that the *A. flavus – Z. mays* interaction involves multiple genes for resistance. These studies also show that there is a strong environmental influence on resistance, which complicates breeding for resistance to *A. flavus* in *Z. mays* (Wisser et al., 2006).

A significant limitation of the current body of genomic work that tackles the *A. flavus* – *Z. mays* interaction, is the tendency to analyze data from the pathogen and the host separately. Although many co-expression networks can be found in the literature, they describe transcription in *A. flavus* or *Z. mays* individually (Sekhon et al., 2013; Asters et al., 2014). Thus, in those studies, interactions between pathogenicity factors in *A. flavus* and resistance genes in *Z. mays* merely denote inferences of association. To gain a better understanding of these interactions in the early stages of the infection, dual transcriptomic analysis of both host and pathogen was undertaken in this study using high depth RNA sequencing. Previous work involved understanding *A. flavus* and *Z. mays* in a small study where there was limited replication reducing the power for making statistical inferences. The study did, however, find that there was significant correlation between pathways in *Z. mays* and *A. flavus* (ref). The experimental design was that of a high-density time series transcriptomic study that allowed the use of casual inference to predict gene regulatory interactions, and to identify key pathways that are active during the early stages of the infection. This provides insight into different gene regulators that are activated at specific times during the infection process, and thus allows for reverse engineering of the entire regulatory pathway. When combined into a gene regulatory network (GRN), the inference of cause-effect relationship between co-regulated genes in pathways within and across species can be comprehensively mapped. Two algorithms were used; GeneNet, a partial correlation/partial variance-based algorithm, and TDARACNE, a time delay algorithm. Both algorithms can determine cause and effect (Opgen-Rhein and Strimmer, 2007; Schaefer et al., 2010, Schaefer et al., 2015). In this work, we used this systems approach to describe the underlying genetics of the molecular interactions between a host and a pathogen in the early stages of infection (SI). Likewise, we provide the first attempt to infer regulatory connections between *Z. mays* and *A. flavus*.

## Materials and Methods

### Growth and Inoculation of Maize

The maize inbred line B73 was grown in the field in Clayton, NC at the Central Crops Research Station at North Carolina State University, during the years 2011 and 2013. Both years were planted on April and grown according to standard practices. Ears were hand pollinated on July 5–8 and covered with a paper bag. *A. flavus* NRRL *3357*, was grown on potato dextrose agar (PDA) and collected from plates with 0.05% (v/v) Triton X-100. In July, a time course study was performed by pin bar inoculating one ear (per time point) from eight maize B73 with *A. flavus* NR3357, and harvesting at 0, 6, 12, 18, 24, 30, 36, 42, 48, 72, 96, 120, and 140 h post inoculation. Samples were frozen in liquid nitrogen, placed on dry ice, and stored at −80°C until RNA was isolated.

### RNA Isolation

Eight kernels per ear were grounded using a mortar and pestle in order to isolate RNA. Approximately one hundred milligrams of ground tissue was homogenized (Virtis, Gardiner, NY, United States) in saturated phenol, pH 6.6 for 2 min. Samples were then dissolved in Tris EDTA buffer, pH 8.0 (ACROS Organics, Morris Plains, NJ, United States), extracted with 5:1 acid phenol: chloroform, pH 4.5 (Fisher), and the RNA precipitated with ice-cold 100% ethanol (ACROS Organics) overnight. Total RNA was purified again with the RNeasy Mini Kit (Qiagen, Hilden, Germany) according to the manufacturer’s instructions. The quality (RIN > 8) and concentration of RNA was analyzed using an RNA Pico chip on an Agilent Bioanalyzer (Agilent, Santa Clara, CA, United States).

### Sequencing

cDNA library construction and sequencing was performed at the Genomic Sciences Laboratory, North Carolina State University. Individual libraries were made from each time point, pooled and run on a single lane. Sequencing was performed on an Illumina HiSeq 2500 platform. The data from the RNA-seq can be accessed at NCBI using the accession (GSE101899).

### Bioinformatics Analysis

For both *Z. mays* and *A. flavus*, mapping, trimming and fastqc quality control of the reads was done with CLC workbench 4.9 (Workbench, 2010). CLC genomics workbench default parameters were used to perform the mapping and trimming similar to previous publication (Musungu et al., 2016). The reference genomes used in the study where *Z. mays* (AGPv3, INSDC Assembly GCA_000005005.5, Apr 2013) and *A. flavus* (JCVI-afl1-v2.0, INSDC Assembly GCA_000006275.1, Jan 2009). Reads that had a total gene count less than 1 were removed from the counts table. The unique reads from CLC genomics workbench were then used with Deseq2 package in R statistical program (Table 1) with the default settings (Love et al., 2014). For contrast selection in DESeq2, SI-1 was considered to be the control. Whereas for the time-course control, the time point samples 0 h were used as the control (Table 1). For *A. flavus*, only samples from SI-8 to 18 where used in the differential expression analysis. The heat maps of the differential expressed genes were analyzed using K-means clustering and hierarchal clustering using Tcluster3 (de Hoon et al., 2004).

**TABLE 1:** The sum of unique reads that aligned to the Maize and *A. flavus* genomes.

Time	Maize	*Aspergillus*
0 h	183546459	16774
4 h	175225669	3700771
6 h	165052452	7223
12 h	191346756	18611
18 h	189130692	119690
24 h	177574520	127124
30 h	149353775	210432
36 h	175423737	765709
42 h	173900923	3029592
48 h	168062408	2716143
72 h	167752678	21747211
96 h	167752678	21747211
120 h	129154376	45150333
144 h	88430362	69915184

### Gene Network Generation

For gene network analysis, the samples fold changes = [gene_i_ – gene_i+n_ (average)] were calculated by using the DESeq2 regularized log transformed data (Supplementary R Script 2) and then using R (Supplementary R Script 1). The R package (GeneNet) was then used for network generation. The criteria for selection into the high confidence networks was a significant partial correlation q-value (0.05) and a significant direction *q*-value (0.05) initially and an additional filtering of the *q*-val.dir < 10^–5^. The gene network also included a “low confidence set” of genes. This set consisted of edges that only had a significant direction *q*-value. The cytoscape 3.0.1 visualization software was used to visualize and display the gene expression network. The edges were depicted as directed graphs to display the causal inference between genes.

## Results

### RNA-seq Analysis During Infection of *Z. mays* Kernels by *A. flavus*

*Zea mays* kernels were infected by pinning with a conidial suspension of *A. flavus*. Pinned kernels were harvested for RNA extraction immediately after pinning (time point 0), and at different time points up to 6 days post inoculation. To analyze gene expression in both *Z. mays* and *A. flavus*, eight biological replicates of each of the time points were sequenced. The Illumina HiSeq reads were separated by organism by simultaneous mapping to both *Z. mays* and *A. flavus* genomes. RNA sequences of *Z. mays* and *A. flavus* were processed by quantile normalization of counts per million of counts uniquely mapping to each gene model. Only unique reads were retained to calculate normalized gene expression as RPKM (Reads Per Kilobase of transcript per Million mapped reads). On average, 20862468.45 reads where mapped uniquely to each of the genomes.

The total amount of sequenced RNA reads in this study, a total of 2303938848 unique reads for maize and 157832429 for *A. flavus*, exceeded what has been reported in other dual RNA-seq studies that have characterized the transcriptomes of host and pathogen simultaneously (Yazawa et al., 2013; Rudd et al., 2015). In the early stages of infection, most of the RNA in each sample was found to be host (*Z. mays*) RNA. This was observed in other dual RNA-seq studies as well and was reported to be simply due to the small number of pathogen cells in the initial inoculum, and the slow initial growth of the pathogen (Musungu et al., 2016). The subsequent growth and spread of the pathogen resulted in increasing the fraction of *A. flavus* RNA in later samples. Interestingly, the number of total unique reads mapping to *Z. mays* did not change significantly from sample to sample until 24 hours post inoculation (hpi) (Table 1). Since the total amount of tissue in each sample was the same for all samples across all time points, the absolute decrease in host RNA reads may also reflect a per gram decrease in living *Z. mays* tissue due to necrosis caused by the pathogen in plant tissue at the later stages of infection, as well as increased amount of pathogen mycelia per gram tissue as the infection progresses.

**FIGURE 1:**
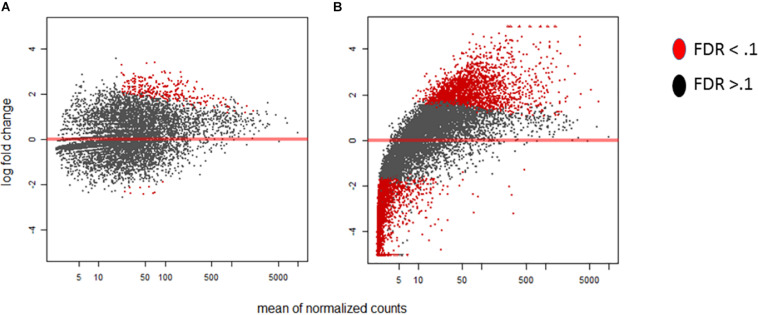
MA-plots were generated for the DESeq2 normalized RNA-seq samples of *Aspergillus flavus*. For this Stage of infection index (SI) analysis, SI7 was used as the background (control). **(A)** MA-plot displays the SI8 treatment relative to SI7. **(B)** MA-plot displays the SI18 treatment relative to SI7. Significant genes are denoted by the red color whereas non-significant genes are colored black.

Within our data we delve into the link between the standard time course vs our stage of infection which represents grouped by *A. flavus* infection dependence. In Figure 1 we highlight stage of infection as opposed to time because it represents a correlation in the activity of transcription for *A. flavus* and *Z. mays* interaction. MA-plots that graphically illustrate the relationship of m-value (log_2_ fold change) vs. absolute expression were used to determine the mean expression distribution and differential expression characteristic of each biological sample (Figure 1). Read counts showed a significant skewing of data, especially for *A. flavus*, that would affect the reliability of differentially expressed gene calling; low counts, especially at early time points, and large dynamic range of RNA-seq data, made log-fold change-based analysis quite noise-prone (Love et al., 2014). DESeq2 regularized log normalization was used during the analysis of the differential expression of genes to reduce the amount of false positives that could be present due to low counts. Additionally, genes that had a total count less than 10 could be removed from the analysis. Consequently, the *A. flavus* transcripts from the early stages of infection (SI1–6) were excluded from the analysis because of the low overall reads. Thus, SI7 was used as the denominator in calculating log fold changes and differential gene expression for *A. flavus*. Even though crucial, many of these early infection stages, had too few overall counts to be considered reliable estimates of relative expression for the pathogen. Similar observations have been reported in other host-pathogen studies (Teixeira et al., 2014).

### Infection Index and Principle Components Analysis

**FIGURE 2:**
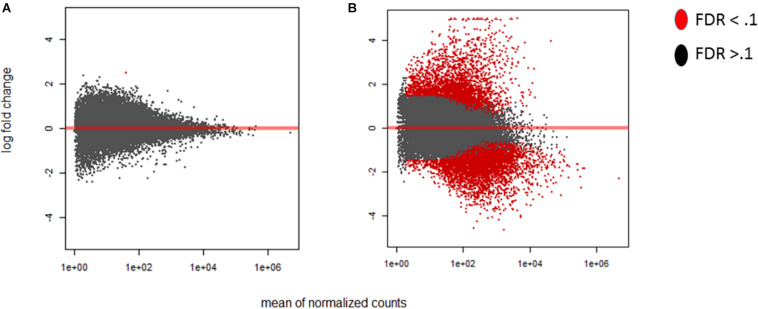
MA-plots were generated for the early and late maize infection stages. The MA-plots illustrate the distribution of the log fold change of the differentially expressed genes. Genes that are significantly differentially expressed are highlighted in red, while those that are not, are displayed in black. For each of the graphs the *Y* axis shows the log fold change, and *X* axis shows the mean normalized counts. The fold change is displayed for the stage of infection index (SI) data to determine the distribution effect on differentially expressed genes in maize. **(A)** SI2 compared to the control SI0. **(B)** SI18, the latest stage, compared to SI0. Differentially expressed genes were determined using DESeq2 (False discovery rate; FDR < 0.1).

There was noticeable variability in the progress of infection over time, with significant discrepancies occurring between 12 and 42 hpi. Not all time points in replicate experiments overlapped in terms of the ratio of *A. flavus* to maize RNA, indicating that our time-ordered samples may be out of order in terms of the stage and progression of infection and disease development. Given the presence of a significant number of outliers in our data, outliers that are typically ignored during differential expression analysis, we developed an additional index to better gauge the progress of infection. The index was built using the ratio of *A. flavus* to *Z. mays* unique RNA, rather than using the time of tissue collection, as the sole criterion to assess infection progress. Consequently, the individual biological replicates were reordered and grouped into 18 Stages of Infection (numbered SI1 to SI18) based on similar infection index values. This reordering was used alongside a time-based ordering of data for subsequent normalization and downstream analyses of the experiments. The effect of this reordering reduced the gene expression variance among replicates significantly. In fact, using the stage of infection index, the maize MA-plots revealed a large change in differential expression relative to the early stage of infection (SI 0 compared SI18 in Figure 2). The time course ordered samples also showed a significant difference in expression as early as 4 h after inoculation. Using the stage of infection index data, it took a few stages before detecting significant differences in expression in maize. For example, SI 2-4 had 36 total genes that were found to be differentially expressed. The difference between time ordered data and stage of infection data is likely resulting from variability in pathogen success at very early time points, leading some biological replicates to reach advanced stages of infection more quickly than others.

**FIGURE 3:**
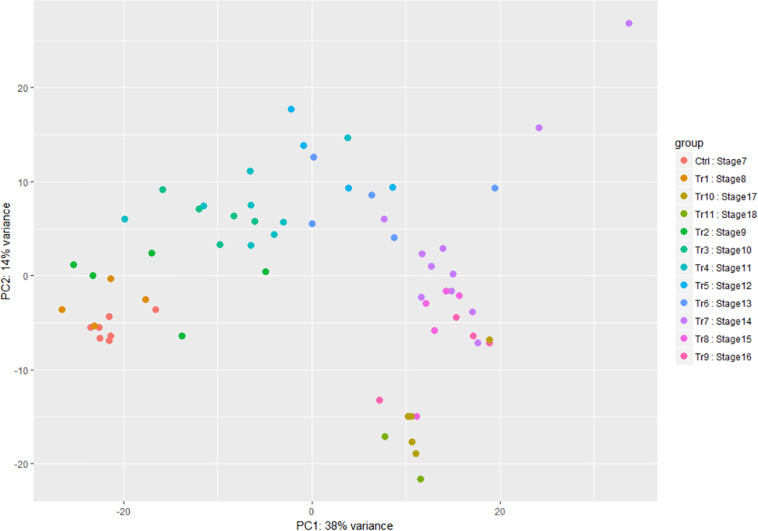
Principal component analysis of the *A. flavus* transcriptomic data. SI7 to SI18 were used to observe variation between samples. Each circle in the graph represents a RNA-seq sample and each color represent a stage of infection (Tr represents Treatment and Ctrl represents Control).

When time points and stages of infection were both analyzed using Principal Component Analysis (PCA), there was some difference between replicates of samples using either grouping, but replicates of the mid to late infection stages, SI–7 to 15, were more tightly clustered together when grouped by stage (Figure 3). Further analysis of the two components revealed a similarity of variance in the components for SI7 and SI8, which are the stages where the transcripts of the pathogen begin to accumulate significantly. Moreover, SI9 and SI10 shifts had similar variances in the components based on PCA analysis. Therefore, SI progression was accompanied by an increase in the *A. flavus*/*Z. mays* expression ratio.

This analysis also showed that, for both host and pathogen, most of the variation in the different biological replicates occurred between samples collected at the same hpi. For *A. flavus*, the clustering of the experiments grouped by SI was more consistent, and tight clustering of experimental replicates was observed. Additionally, the time ordered data displayed a variance where most of the variance was explained by the *x*-axis component. The hpi time points had multiple samples with similar overlap between samples. For example, hpi 0, the point after inoculation which was considered to be the control, shared a tight cluster and overlapped with hpi 4 and 6. Similar results were found with the later timepoints. For instance, this is reflected in the difficulty to distinguish transcriptomes from the 5 and 6 days post inoculation samples. Similarly, the maize component data was ordered by time with multiple samples showing overlap between different hpi.

### Differential Expression Analysis for *Z. mays*

The differential expression analysis of *Z. mays* was performed utilizing the (Stage of Infection) SI index to organize the samples for the downstream analysis. Multiple genes were found to be differentially expressed in at least one infection stage in *Z. mays* when DESeq2 was used to compare later infection stages to SI-1 (Supplementary Table S1). It should be noted that the samples at the early stage of infection showed very low pathogen RNA levels. However, at SI-3, there was an increase in both the number of significantly differentially expressed up/down regulated maize genes (7 genes) as well as higher levels of pathogen RNA. The largest increase from SI-2 in terms of the number of detected differentially expressed genes did not occur until SI-6 when the activation of multiple genes that are known to be involved in the resistance to *A. flavus* was observed (Table 2). The one gene (GRMZM2G175574) detected in SI-2 that had a significant differential expression (*p*-adj < 0.1) probably indicating a lack of response to the pathogen at this stage (Table 2). For SI-3, there were only 7 additional differentially expressed genes (Table 2). Genes in *Z. mays* kernels began to show significant differential regulation around 30 hpi when the datasets were arranged by time, or at SI-7 when evaluated by infection index. Notably, this is the same stage at which *A. flavus* RNA became abundant enough to be analyzed reliably. At the crux of the infection (144 h, infection stage 18), 2345 maize genes were significantly up-regulated, and another 4057 were suppressed. Many of these genes were components of pathogen resistance pathways.

**TABLE 2:** Differentially expressed genes of maize at each of the Stages of Infection.

Stage of Infection	Differential expression count	Up	Down
*SI 2*	1	1	0
*SI 3*	7	6	1
*SI 4*	28	24	4
*SI 5*	50	50	0
*SI 6*	359	279	80
*SI 7*	39	32	7
*SI 8*	159	56	103
*SI 9*	235	200	35
*SI 10*	470	270	200
*SI 11*	872	664	208
*SI 12*	2173	1611	562
*SI 13*	1851	1309	542
*SI 14*	5157	3499	1658
*SI 15*	4267	2094	2173
*SI 16*	6274	2910	3364
*SI 17*	9416	4539	4877
*SI 18*	6402	2345	4057

### Gene Ontology Enrichment Analysis Using SI for *Z. mays* and *A. flavus*

Functional enrichment analysis was used to reveal pathways in both the host and the pathogen, involved in the interaction between the two organisms. Analysis of data from the early infection stage (SI-2) identified the *Z. mays* gene GRMZM2G175574 to be the only differentially expressed gene in the SI-2 dataset (Supplementary Table S1) therefore no geno ontology analysis was possible during those early SI’s. When observing the groups of SI genes found in the data set many of the common resistance genes were found. These genes included the late embryogenesis abundant 3 gene (GRMZM2G072890), sucrose synthase 4 (GRMZM2G008507), as well as multiple chitinase-related genes.

At SI-9, 235 genes were differentially expressed. These included key marker genes involved in pathogen resistance such as 12-oxophytodienoate reductase 2 (GRMZM2G156712), a gene involved in the biosynthesis of jasmonate that has been implicated in resistance to necrotrophs (Robert-Seilaniantz et al., 2011; Mur et al., 2012, Lyons et al., 2013), and endochitinase A (GRMZM2G051943), an antifungal enzyme implicated in resistance to *A. flavus* (Dolezal et al., 2014). Later stages showed a dramatic increase in the number of differentially expressed maize genes at SI-12, and a corresponding increase in the number of *A. flavus* genes at the final stage SI-18. Common *A. flavus* resistance gene markers were observed in both mid- and late-infection stages. For *Z. mays*, there was also a considerable overlap between genes in the late stages. Notably, only one of the early stages genes, the uncharacterized GRMZM2G175574, was still differentially expressed at the later stages of infection. The overlap that was observed from SI-10 to SI-18 consisted of 60 genes including resistance genes, such as (GRMZM2G117942) and (GRMZM2G117971) which are chitinases, (GRMZM2G475059) and (GRMZM2G156877) glutathione S-transferase, and (GRMZM5G894619) 1-Aminocyclopropane-1 carboxylate synthase.

GO-term enrichment analysis of maize genes differentially expressed at SI-18 revealed multiple pathogen resistance pathways. Up-regulated genes detected by SI-18 included Endochitinase A (GRMZM2G051943) and Coronatine-insensitive protein 1 (GRMZM2G151536) (Naumann et al., 2009; Hawkins et al., 2015). Several of the genes were linked to the following GO pathways: GO:0055114- oxidation-reduction process, GO:0016491-oxidoreductase activity and GO:0004497-monooxygenase activity. Analysis of this stage of infection also revealed multiple unique genes that were not detected in any of the earlier stages. In fact, 1890 genes were unique to SI-18. Many of these genes did not significantly correlate to any known pathways. Fifteen of the genes that became active between S-18 and S-I9 are known resistance genes previously implicated in resistance to *A. flavus.* Most of the GO-terms that were highlighted at these later stages of the infection were primarily involved in responses to stimuli and in the production of secondary metabolites. This could be linked to the progression of *A. flavus* at these late stages. By SI-18, down regulated defense GO-terms began to appear. These included known resistance genes like lipoxygenase (GRMZM2G109056), PR-10 genes (GRMZM2G112524, GRMZM2G112538, GRMZM2G112488) and chitinase (GRMZM2G145518) which are involved in signaling and combating fungal infection (Chen et al., 2010; Christensen et al., 2015, Hawkins et al., 2015). Many processes were initially upregulated in the early stages of the infection index and appeared to peak in the later (SI12–18) (Hawkins et al., 2013).

Although only a few transcripts of the pathogen genes were detectable at the early stages of infection (SI1–6), many shared orthologs with developmental genes found in other fungi. For instance, the most abundant *A. flavus* gene detected at SI-1 was (AFLA_090780), the translation elongation factor EF-1 alpha subunit. This gene retained one of the highest mean values for absolute expression throughout the study, although it was not significantly differentially expressed. This makes this gene a useful marker to assess the relative abundance of *A. flavus* in a sample. The total abundance of reads for *A. flavus* in the complete data set was 169272008 reads, most occurring in the later infection stages.

**FIGURE 4:**
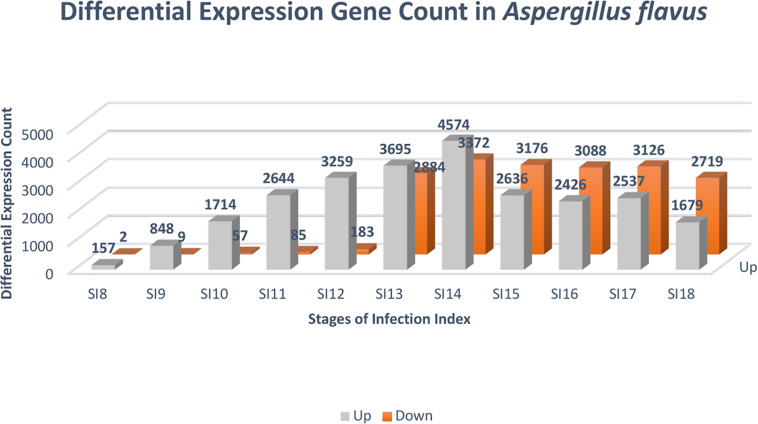
Differential expression analysis was conducted with DESeq2 to identify genes that were significantly differentially expressed at the stages of infection. The color gray denotes genes that were up-regulated, and the color orange indicates genes that were down-regulated. The FDR cutoff used to call differentially expressed genes was (*p* adjusted value < 0.05).

Due to the low coverage of *A. flavus* reads at early stages of infection, SI-1 to 6 were not included in the analysis for differential gene expression in *A. flavus*. SI-7 was chosen as the starting point for differential gene expression analysis rather than using RNA from a conidial suspension, because it is more likely that the infection started by already germinated conidia. Throughout (SI 8-18), shifts in gene expression in *A. flavus*, reflected more up-regulation of genes rather than down-regulation (Figure 4); 159 *A flavus* genes were found to be differentially expressed (157 up-regulated and 2 down-regulated) at SI-8 (Table 3). Many of the genes observed at this initial stage involved primary metabolism and were enriched for primary metabolism - related GO terms. Gene Ontology pathways involving the carboxylic acid metabolic process GO:0019752 and the monocarboxylic acid metabolic process pathway GO:0032787 were significantly enriched. At SI-9, 857 *A. flavus* genes were found to be differentially expressed and GO-TERMS involving nitrogen metabolism, such as the nitrogen compound metabolic process GO:0006807, proteolysis GO:0006508, organ nitrogen compound metabolic process GO:1901564, and the cellular nitrogen compound biosynthetic process GO:0044271. At SI-10 there was a large increase in differentially up-regulated *A. flavus* genes detected in the data (Table 3). Interestingly, this stage was marked by a significant increase in the expression of aflatoxin cluster genes. The global regulators *AflR* and *AflS* were detected within this dataset to be differentially expressed. There were also biological processes that were enriched at SI-10 including GO:0032502 (developmental processes), GO:0006897 (endocytosis) and GO:0043436 (oxoacid metabolic process). As *A. flavus* progressed at later SI’s, there was significant overlap with biological processes reported in other studies involving terms such as GO: 0005975 (carbohydrate metabolic process), GO:0019538 (metabolic processes), and GO:0016192 (vesicle-mediated transport) (Bai et al., 2015).

**TABLE 3:** Differentially expressed genes identified following the DESeq2 analysis of stages 7 to 18.

Stages of Infection *Aspergillus*	Total	Up	Down
*SI8*	159	157	2
*SI9*	857	848	9
*SI10*	1771	1714	57
*SI11*	2729	2644	85
*SI12*	3442	3259	183
*SI13*	6579	3695	2884
*SI14*	7946	4574	3372
*SI15*	5812	2636	3176
*SI16*	5514	2426	3088
*SI17*	5663	2537	3126
*SI18*	4398	1679	2719

### Clustering Analysis

Clustering is widely used to determine correlations between pathways. Therefore, differentially expressed *A. flavus* and *Z. mays* genes were clustered using K-means. The genes clustered into 100 groups capturing “unions” and “intersections” between *Z. mays* and *A. flavus*. It is to be noted that *Z. mays* genes reported to be involved in disease resistance often clustered together, even when their presumed biological roles are different. Furthermore, K-means clustering revealed the polygenic nature of the interaction between *Z. mays* and *A. flavus*.

Further analysis of the clusters uncovered maize defense genes such as (GRMZM2G156006) *AP2*/*EREBP* transcription factor, (GRMZM2G088765) Peroxidase 54 and other uncharacterized genes. Moreover, one of *Z. mays* co-clusters included genes that were initially up-regulated, but later displayed depleted levels of expression (Supplementary File S1). This group of genes may comprise resistance genes that are part of the earlier responders to *A. flavus* infection and to the exposure to mycotoxins.

**FIGURE 5:**
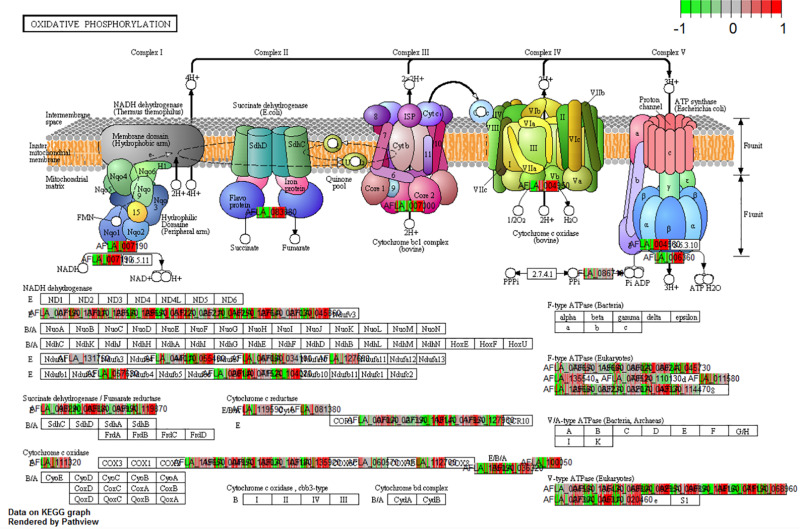
KEGG pathway analysis on the different Stages of Infection (SI) was done to observe the enrichment of pathways in the pathogen *in vivo*. The current pathway was a significant enriched and differentially expressed within the dataset (*q*-val < 0.1) when comparing SI8 vs. SI7.

**FIGURE 6:**
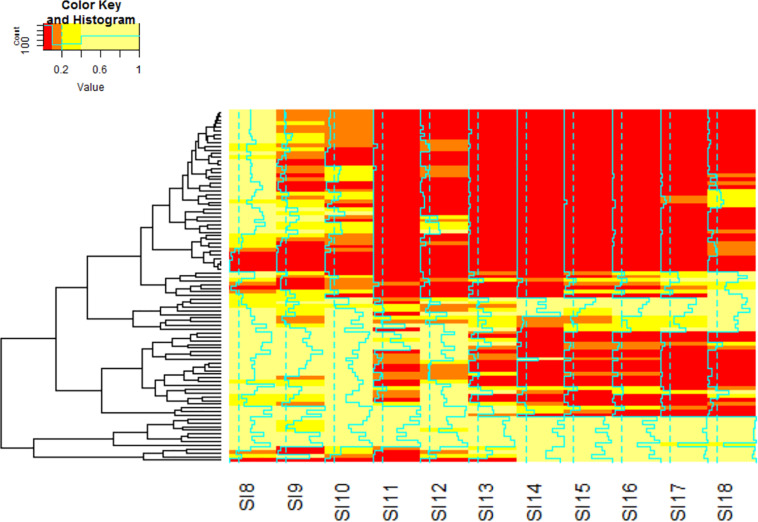
Hierarchal clustering analysis of the KEGG Pathways by *q*.value was performed to identify the pathways that are significantly involved in the interaction. The color red denotes genes with the lowest *q*.value. Orange indicates genes with moderate significance within the data set. Yellow indicates genes that do not exhibit significant differential expression. The blue lines through each stage indicate the strength of the clustering by the *q*.value. The order of the matrix is stored in Supplementary Table S1.

The analysis of differential gene expression in *A. flavus* with K-means clustering revealed shifts in gene expression patterns from SI-7 to SI-18. The *A. flavus* transcriptome transitioned from an inactive state in SI-6 to an active state in SI-7 as revealed by a dramatic shift in gene expression. Multiple pathways were initially activated and later down-regulated (Supplementary Table S3). Further analysis revealed multiple pathways that were significantly enriched and down-regulated; the oxidative phosphorylation pathway (Figure 5), multiple genes involved in RNA transport, and genes involved in ribosome biogenesis, were among the pathways that were significantly down-regulated (Supplementary File S1 and Figure 6). Interestingly, the oxidative phosphorylation pathway includes genes that have previously been implicated in the biosynthesis of mycotoxins (Grintzalis et al., 2014).

In some cases, gene expression was highly elevated during the later stages of infection. For example, cluster 32 contained *A. flavus* and maize genes that were activated at the mid stage of infection. The *A. flavus* arsenal of hydrolytic enzymes, such as (AFLA_007720) pectin lyase, (AFLA_124660) pectin lyase precursor and (AFLA_023340) pectinesterase precursor, were found to be upregulated at the later stages of infection. This cluster also surprisingly contained pectinesterase 11 (GRMZM2G070913) of maize.

The aflatoxin cluster gene *AflR* clustered with aflatoxin cluster genes *AflMA*, *AflQ*. Our data did not find all the aflatoxin cluster genes to be located in a single cluster in the analysis. The jasmonate biosynthesis 12-oxophytodienoate reductase 2 maize gene (GRMZM2G000236) was included in the same cluster, and appeared to be co-expressed with *AflR*, *AflMA* and *AflQ*. Additionally, that same cluster contained the maize genes for chalcone synthase (*C2*), an *AP2*/*EREBP* type transcription factor (GRMZM2G159592), and multiple reactive oxygen species domain containing genes.

### KEGG Analysis for *Z. mays* and *A. flavus*

**FIGURE 7:**
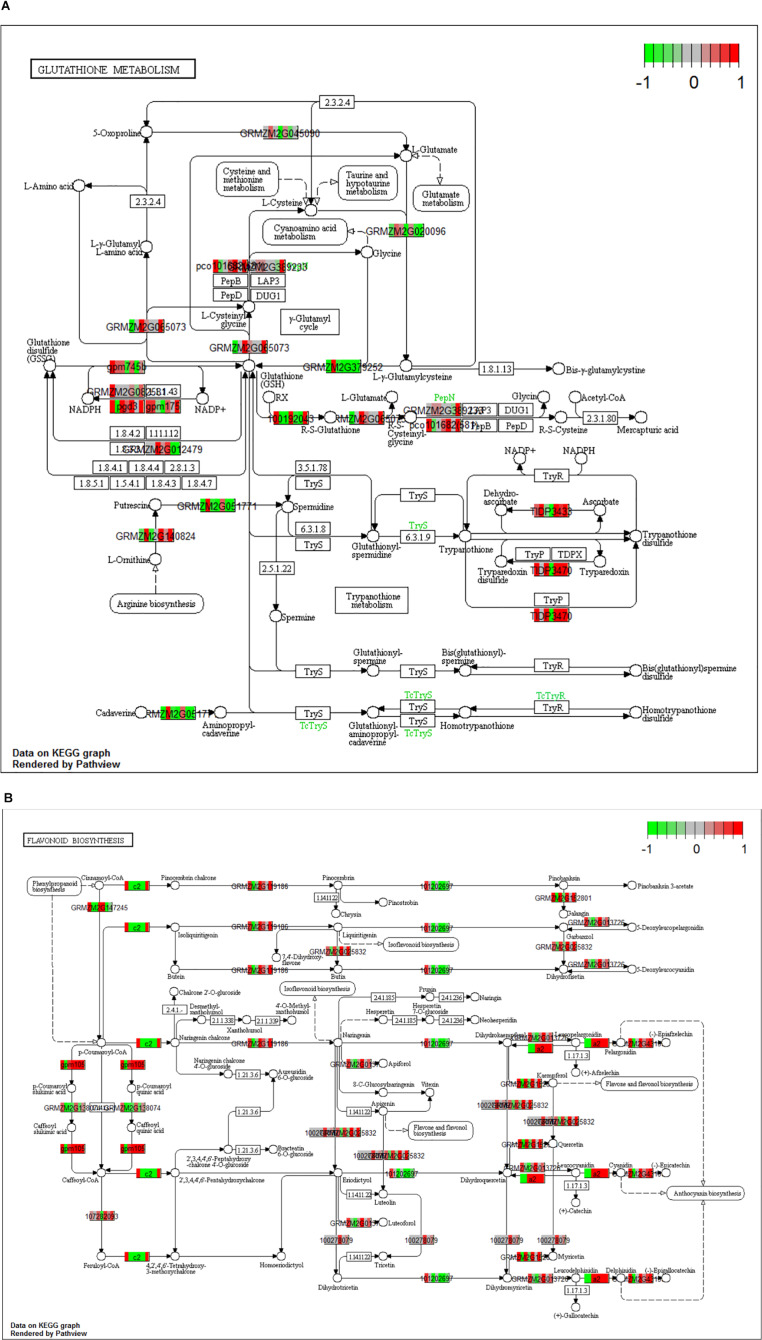
**(A,B)** Kegg analysis was done on the Stage of Infection (SI) 6 index to determine pathways that were significantly enriched within the *Zea mays* data set. For the boxes that are white indicate genes where it was unable to find a match in the pathway. **(A)** Signifies the gluthatione pathway (KEGG pathway id: ko00480) and **(B)** signifies the flavonoid pathway (KEGG Pathway: ko00941). The cutoff for a significant pathway was signified with a (qval < 0.1) for each of the SI.

When the dual transcriptomic SI data was analyzed using Kegg Pathview package in R, the first pathway found to be significantly enriched was the DNA replication pathway for *Z. mays* (Luo et al., 2017). In the earlier stages, there was not a significant enrichment in the pathways usually involved in resistance to *A. flavus*. The detection of these enriched pathways did not occur until SI-6 when the flavonoid biosynthesis and the glutathione metabolism pathways were found to be significantly enriched (Figure 7). This result was also reported in other GWAS metabolic analysis studies involving the *A. flavus* and *Z. mays* interaction (Tang et al., 2015; Warburton et al., 2015). Our results also agree with the identified pathways, like amino acid metabolism, which was found to be significantly enriched as early as S-I5 in the data set (Tang et al., 2015). The ethylene biosynthesis pathway was found not to be significantly enriched in the KEGG pathway analysis, whereas the building blocks for the pathways regulating cysteine and methionine metabolism were enriched in the GO-analysis for the data set. Furthermore, α-linolenic acid metabolism (jasmonate) was significantly enriched at the SI-14.

Further investigation using KEGG pathway analysis for *A. flavus* revealed additional pathways that were initially activated and later down-regulated (Luo and Brouwer, 2013). When SI-9 was analyzed, pathways such as the biosynthesis of secondary metabolites, biosynthesis of amino acids and carbohydrate metabolism were up-regulated and significantly enriched. The aflatoxin pathway was found to be significantly enriched in the SI-10 data set. The analysis also revealed multiple pathways that were significantly up-regulated and enriched; the oxidative phosphorylation pathway (SI-10-18), and multiple genes involved in RNA transport (SI-10-18). One pathway that was significantly enriched in the analysis of the early stages of infection and that was later depleted, was the ribosome biogenesis pathway (Figure 6). Interestingly, it was the only pathway to deplete significantly in the later stages of infection.

### Interactome Analysis

The differentially expressed genes for *A. flavus* and *Z. mays*, were then analyzed in their respective predicted protein interactomes to allow for inference for of novel protein protein interactions (Musungu et al., 2015; Szklarczyk et al., 2015). There was a total of 1451 proteins that were found to be in the *Z. mays* interactome. Some of the highest connective proteins that were identified was GRMZM2G030299 (Protein-ribulosamine 3-kinase chloroplastic). This transcript was highly down-regulated in the DESeq2 analysis, with a log fold change of −0.54 (DeSeq2 Fold Changes). This was followed by GRMZM2G097878 serine-threonine protein kinase, which had 299 connections. The corresponding gene was also down-regulated in the data set. The next gene GRMZM2G004356 (transcription factor *UNE12*-related) had 254 connections and was up-regulated through the initial stages of infection and down-regulated from stage 13 to 18. At each stage of infection, the differentially expressed genes had different amounts of interacting partners in the interactome of *Z. mays*. The largest subnetwork for the infection study was produced from the later stages that had the largest number of differentially expressed genes. Additionally, analysis from the PiZeaM disease subnet differential identified 400 common targets that are predicted to be involved in biotic response. From the hormonal response network, 23 genes were identified within the interactome with 31 interacting partners. With many of the genes involving reactive oxygen species pathways, heat shock proteins and jasmonate pathway genes. The multiple proteins identified and pathways determined demonstrate the polygenic nature of the resistance to *A. flavus*.

With *A. flavus*, the network was highly dense including 927 differentially expressed genes with 3584 interacting protein partners. The network contains proteins that are involved in development, which were also shown to play a role in mycotoxin and aflatoxin production. Networks also contained carbohydrate and nitrogen metabolism subnetworks. The *A. flavus* subnetworks followed a pattern similar to that seen in *Z. mays* subnetworks, where the connectivity or interacting protein partners increased over time.

### Gene Regulatory Network Analysis

**FIGURE 8:**
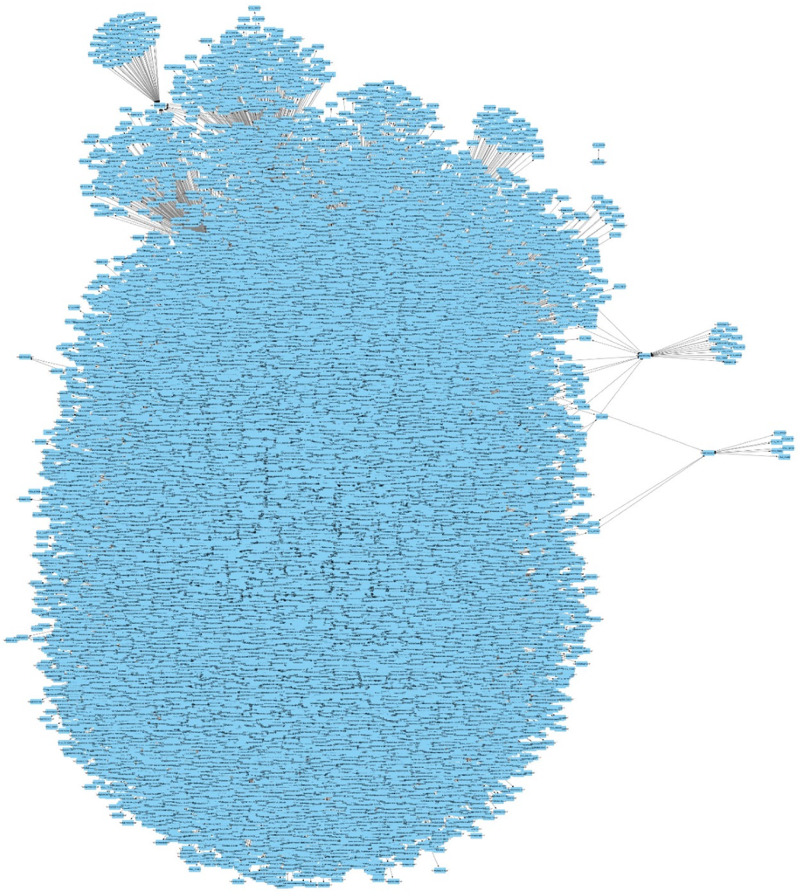
Gene regulatory inference using the Stage of Infection ordered data to observe partial correlated interactions within the network constructed from combined *A. flavus* and *Z. mays* data.

The multiple time points allowed the generation of a stage of infection index for the transcriptomic study. The GeneNet (Schaefer et al., 2015) module was utilized to develop a partial correlation network where both host and pathogen transcriptomes were present. The criteria for cutoff in edge selection was done using familywise false discovery corrected *p*-values (*q*-val and *q*-val.dir < 10^–5^) (Figure 8). As expected, this produced a large amount of edges which agrees with what was reported in many gene regulatory network identification studies. Therefore, to determine if the edges were significant in the network connectivity, genome wide association data, and quantitative loci information involved with resistance to *A. flavus* were used to mine for significant genes in the directed network. The edges for the network can be retrieved using (Supplementary Code S1). The network was generated from 47,801 genes. We did not find any significant enrichment within the unused genes from the transcriptome for both *Z. mays* and *A. flavus*. This network adds to previous work that only utilized gene co-expression from Pearson Correlation and utilizes partial correlation to determine significant genes within the network.

First, the differentially expressed genes were evaluated for hub enrichment to determine connectivity. The connectivity (or Degree) has previously been shown to be an indicator of biological importance (Horvath and Dong, 2008). Second, publicly available data from chip-seq and GWAS (genome-wide association studies) studies was used to determine coverage of resistance marker genes and transcription factors (Bolduc et al., 2012; Eveland et al., 2014, Tang et al., 2015). The connectivity of *A. flavus* genes highest interactors were found to include genes from the up-regulated KEGG pathway involved in endocytosis (Supplementary Table S4). Of the *Z. mays* genes found in the network to contain the most connection was a predicted translation initiation factor SUI1 (GRMZM2G017966) which according to qteller (Schnable, 2014) has strong expression in the pericarp of the tissue (Table 4). When looking at the DESeq2 log_2_Fold changes, the gene was activated in the earlier stages (SI-2-9), but expression was not detected at later stages of infection. Early expression was observed for many of the top 20 highly connected genes within the network. Included in this group of highly connected genes were *RPM1*, *SAM* and a leucine rich repeat receptor gene previously implicated in resistance.

**TABLE 4:** Connectivity/Degree for genes identified in the host pathogen network. Gene ID refer to the maizegdb annotated gff3 file.

Gene ID	Annotation	Node1	Node2	Total
*GRMZM2G017966*	Uncharacterized protein	3112	14556	17668
*GRMZM2G073308*	Uncharacterized protein	6009	10045	16054
*GRMZM2G122563*	ribulose bisphosphate carboxylase	7535	8212	15747
*ESR3*	embryo surrounding region	1740	13816	15556
*GRMZM2G105085*	Porin_dom	6646	8867	15513
*PSBB*	Photosystem II CP47 reaction center protein	15258	59	15317
*GRMZM2G464891*	Uncharacterized protein	13153	2079	15232
*GRMZM2G063438*	S-adenosyl-L-methionine-dependent methyltransferases	4566	10544	15110
*GRMZM2G397788*	(RPM1, RPS3) NB-ARC disease resistance protein	11611	3241	14852
*GRMZM2G119717*	leucine-rich repeat receptor-like protein kinase family protein	7189	7651	14840
*GRMZM2G358180*	PS_antenna-like	10817	3880	14697
*GRMZM2G075386*	Acanthoscurrin-2	4732	9860	14592
*RBCL*	Ribulose bisphosphate carboxylase large chain	14511	45	14556
*GRMZM2G096792*	PSI_PsaC	5689	8816	14505
*GRMZM2G445961*	predicted pleiotropic drug resistance protein	11950	2170	14120
*GRMZM2G116137*	Transcription factor PCF6	6664	7184	13848
*GRMZM2G404453*	Uncharacterized protein	10778	2835	13613
*GRMZM2G148605*	Flavin_mOase	7532	5815	13347
*GRMZM2G370044*	Uncharacterized protein	10126	3190	13316
*NDHG*	NAD(P)H-quinone oxidoreductase subunit 6, chloroplastic	13228	70	13298

The transcription factors that were present in the network are the *WRKYs*, *AP2*, *MYB* and *NAC* that were previously found in the gene ontology enrichment analysis. The largest *WRKY* transcription factor found within the network was (GRMZM2G383594) which has already been implicated in resistance studies with other pathogens as well as with *A. flavus* (Gao et al., 2014) (Supplementary Table S3). There was also *WRKY* genes that were targeted by *A. flavus* genes; the network contained 107 different WKRY genes based on UniProt Consortium (2015).

**FIGURE 9:**
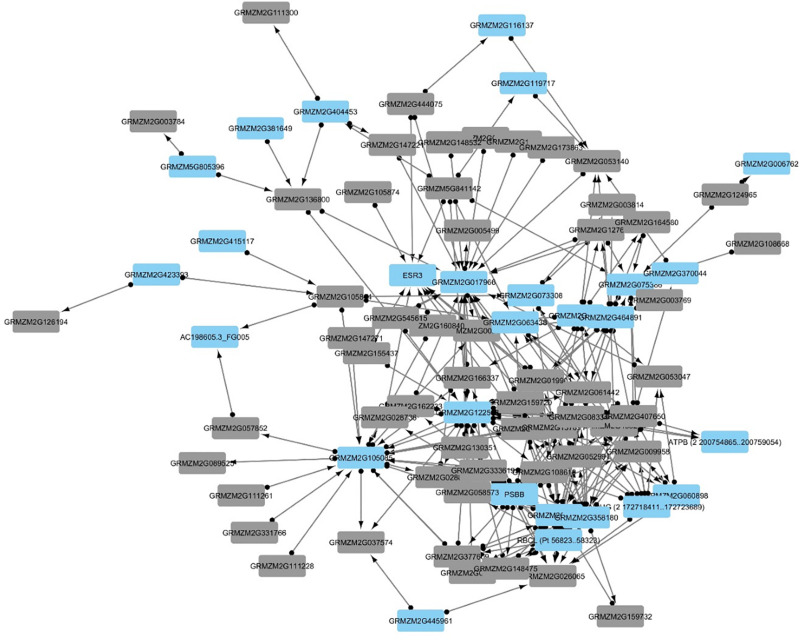
The network represents 83 genes, which were inferred from the Stage of Infection transcriptomic analysis. Since the network was large, only edges with a FDR < 10^– 5^ were used in building the network. Additionally, the edges in the network connect genes that were found to be partially correlated and that have a downstream effect. The blue nodes indicate genes that have not been described in previous aflatoxin/ear rot- related genome wide association studies (GWAS). Gray nodes indicate genes that have been previously associated in resistance from GWAS to *A. flavus*.

*Z. mays* genes identified from previous GWAS analysis studies formed a network where multiple GWAS genes were correlated with differentially expressed genes (Figure 8). Hub genes were likely to occur more for the target genes compared to the source genes. This indicates that the network can capture hypothetical upstream targets as well as downstream targets. The latter can be used as likely candidates for marker assisted breeding, Further analysis was performed utilizing the genes identified through GWAS studies involved in resistance to *A. flavus*. This list contained 66 genes that were originally found to be associated with aflatoxin resistance in grain (Warburton et al., 2015). When mining the differentially expressed genes, 56 genes were inferred to be involved with 30 other maize genes. The gene from the GWAS study with the largest connectivity within our total network was GRMZM2G162233, which is still predicted to be uncharacterized according to the latest maize genomics data. It however, shares orthology with AT1G18730 which is predicted to be a NAD(P)H gene. Overall the network analysis was able to capture 56 genes out of the 66 highlighted in recent GWAS studies (Figure 9).

## Discussion

**FIGURE 10:**
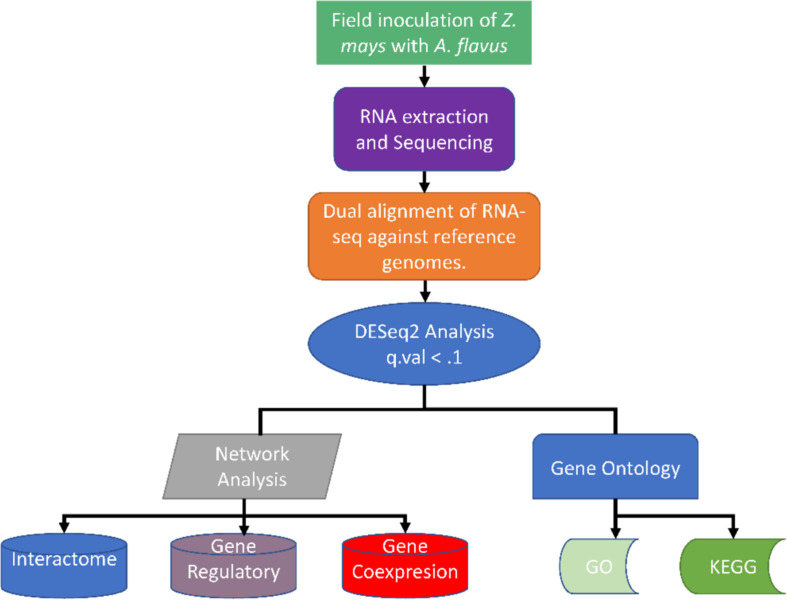
Summary of the different methods used for analyzing the dual RNA-seq study for *Zea mays* and *Aspergillus flavus*.

To uncover some of the complexity of the interaction between *Z. mays* and *A. flavus*, a systems biology approach was undertaken (Figure 10; Saito and Matsuda, 2010). This has been demonstrated to beneficial in reusing available data build complex models and networks (Brandl and Andersen, 2017; Rodenburg et al., 2018). In addition, a classical qualitative epidemiological approach of categorically ranking samples by the detection of *A. flavus* was utilized for the first time to group samples for differential expression analysis and gene regulatory network inference. This was beneficial in that the traditional time series analysis assumes that the host pathogen interaction is theoretically synchronous upon the point of infection (OBrian et al., 2003; Chang et al., 2007). However, our data for *Z. mays* and *A. flavus* suggested that that is not the case as revealed by the PCA analysis; the samples showed variability with confounding factors which are common *in vivo* studies, justified the development and adoption of the novel stage of infection index. This was noted upon observing the reads values for *A. flavus* genes that were identified in the initial time points and stages of infection.

The amount of *A. flavus* genes detected in the study was highly variable depending on the sample, and it ranged from 158 to 11961 genes. This is probably due to the low levels of detected pathogen RNA in the earlier stages of the infection, when only highly expressed genes could be detected. The subset of *A. flavus* genes in these samples represent the pathogen beginning to be able to establish itself in the kernel tissue. This has been attributed to background amounts of pathogen originating from the field or the soil used in those experiments. For example, the pathogen *Moniliophthora perniciosa* was detected with less than 1000 transcripts using RNA-seq libraries with close to 80 million reads (Teixeira et al., 2014).

Our data also shows a unique increase/decrease in the amount of gene expression activity between the host and pathogen. The progressive increase of unique *A. flavus* RNA reflected the progression of disease; the increase of the relative amount of *A. flavus* RNA with time indicates fungal growth. On the other hand, the level of *Z. maize* RNA remained unchanged and eventually dropped. This is likely due to significant cell death from necrosis, which is one of the reported outcomes of infection by *A. flavus* (Dolezal et al., 2013).

Our approach of using stages of infection captures the divergent properties observed in the transcriptomes of the host and the pathogen during the early, mid and late stages of infection based on our study of early infection. The gene regulatory network approach was then further utilized to infer causal relationships between genes within host and pathogen, and between host and pathogen (Guo et al., 2016; Banf and Rhee, 2017). This novel approach expands on the previous Pearson correlation analysis which studied the two organisms independently, and revealed a significant dependence between the interactions of the two organisms (Musungu et al., 2016; Zheng and Huang, 2018). In addition, genes were modeled into correlative interactions groups using linear correlation methods. Connections between expression patterns and expression clusters in transcriptomes were drawn by mapping them to biological functions. Similar approaches were previously used in research were transcriptomes of hosts and pathogens were studied separately (Musungu et al., 2016). Moreover, we examined correlations and clusters between host and pathogen genes that may indicate inter-organismal interactions of biological functions or pathways. This study captured all known regulators and identified several new regulators that activate their targets at time points not covered in previous studies.

An *A. flavus* and *Z. mays* gene regulatory network, containing 47,801 genes and multiple regulatory connections, was constructed to observe causal relationships. The network can be used to study many pathways not directly involved in host-pathogen response, but that are active in this 7-day time series. We focused on subnetworks and key pathways involved in resistance in *Z. mays* and in affecting the mechanisms of pathogenicity that *A. flavus* utilizes to infect susceptible hosts. Our initial selection of the SI-1 point is key for capturing possible circadian genes that were likely to be affected throughout the SI’s. This was confirmed with the maize KEGG analysis. This was also reflected by the significance of down-regulation of multiple *Z. mays* circadian rhythm genes.

Upon assessing the differential expression data, the *A. flavus* transcriptome seemed to have a progressive effect on the *Z. mays* transcription. A similar conclusion was reached in a previous study using Pearson correlation exclusively (Musungu et al., 2016). Until SI-2, *A. flavus* did not have any differentially expressed genes that were up-regulated. This could have been due to *A. flavus* utilizing preformed transcripts as it has been suggested in other studies with different *Aspergillus spp.*(van Leeuwen et al., 2013). SI-6 was when an increase in transcriptional activity in *A. flavus* was noted. Moreover, multiple resistance marker genes such as PR1, OPR2 and PR10 were activated by SI-4. Although none of these marker genes were found to be statistically significant in the differential expression analysis, they were captured within the gene regulatory network. The GRN was able to visualize genes activated at these early stages for *A. flavus*, with many of these genes serving as downstream targets within the network.

The aflatoxin cluster was significantly differently expressed for many of the genes in the cluster. However, we were unable to capture the complete aflatoxin cluster in the GRN network. This is most likely due to limitations of the genes that were kept for the analysis as well as the partial correlation significance values. This could be also due to the complexity effects on the expression of the aflatoxin pathway as shown in studies involving temperature, pH and carbohydrates (Yu et al., 2004; Amare and Keller, 2014, Grintzalis et al., 2014; Medina et al., 2017, Gilbert et al., 2018). There is also the possibility when dealing with partial correlation that the connection was lost due to hidden confounders within the dataset. *AflR* was the only aflatoxin cluster gene not to be detected during the gene regulatory analysis. We however, found the rest of the cluster to be present including *AflS* which has been implicated to be a co-regulator in the cluster.

A shift in many of the pathways, including starch and sucrose metabolism as well as the oxidative phosphorylation pathway, was observed at SI-9. These pathways were down-regulated by SI-18 for *A. flavus*. This likely reflects the abundance of cytochrome P450s that were present in the differentially expressed gene set. The presence of these oxidative stress genes is most likely related to the abundance of reactive oxygen species causing genes, such as the peroxidase (GRMZM2G177792, AC210003.2_FG004, GRMZM2G410175, GRMZM2G408963, and GRMZM2G089982) to be activated in the kernel at this infection stage. Interestingly, it has been reported that in *A. fumigatus*, the loss of cytochrome expression can lead to an increase in pathogenesis *in vivo* (Grahl et al., 2012). This was the case in our study at SI-18. A similar process pattern was noted for *A. fumigatus in vivo* studies involving aspergillosis (Grahl et al., 2012). In our study, this switch in *A. flavus* was probably induced by the activation of resistance genes in *Z. mays*.

Our data also agrees with the previous work showing the importance of the WRKY TF family (Fountain et al., 2015b). For example, WRKY TFs which have been shown to be involved in abiotic and biotic stress, were found in our study targeting multiple jasmonate induced proteins (GRMZM2G020423, AC206425.3_FG002) and heat shock proteins that were conserved in the maize interactome. With our GRN, *Z. mays* genes, orthologous to biological stress pathway genes in *Arabidopsis thaliana*, being also initially targeted by specific *WRKY* genes. Moreover, the induction of *WRKY* TFs affected primarily resistance genes, but *A. flavus* oxidative stress genes such the O-methyltransferase group, lipase, and hydrolase were observed in the network as well. This was interesting because our data showed that the differentially expressed genes such as alternative oxidase (AOX2) and Cytochrome P450 (GRMZM2G147774) to be correlated with the aflatoxin cluster genes. This agrees with previous work involving environments rich in reactive oxygen species and their effect on *A. flavus* (Reverberi et al., 2012; Zaccaria et al., 2015). This subset of the network identified targets that were induced in the expression study and that share functional similarity to genes involved in resistance to pathogens.

In conclusion, this system biological approach utilized the available body of information to determine gene regulatory networks as well as motifs for co-regulated partners. The produced information can improve the broad understanding about early processes that are involved in resistance to *A. flavus*. However, it is important to note that one of the limitation of the data-set was that it was not complete by not encompassing the analysis of every possible gene that was expressed in the transcriptome. It is likely that even though many genes were unable to pass the threshold of detection for selection as a node, they could still make up the motifs with the network intermediates. Another observation made during the analysis of the GRN was that there were not many of the linear relationships typically seen in co-expression analyses given the partial correlation preferential detection of motifs. This is to be expected given the inclusion of multiple variables, once considered to be governed by a common regulator that are now partially explained by multiple regulators. Previously these partial regulations would have been masked using analyses that rely solely on Pearson correlation. To date, this analysis provides one of the first comprehensive transcriptomic dual RNA-seq studies in a plant-pathogen system.

## Data Availability Statement

The datasets generated for this study can be found in the GSE101899.

## Author Contributions

BM conceived the original research plans. SQ, RB, MG, and AF supervised the experiments. GO’B and GP carried out sequencing. BM wrote the manuscript with contributions from all the authors.

## Conflict of Interest

The authors declare that the research was conducted in the absence of any commercial or financial relationships that could be construed as a potential conflict of interest.

## Supplementary Material

TABLE S1 **(A,B)** DESeq2 outputs for all the stages of infection (1 to 18). Differentially expressed genes found in *Zea mays* after using DESeq2.

TABLE S2 KEGG Gene Set Enrichment for *A. flavus*. Pathway enrichment analysis was done for Stages of Infection 8 to 18. Each column has a *q*-val which was considered to be significant (*x* < 0.1).

TABLE S3 WRKY Transcription Factor Network. This data set contains the subnetwork gene net for the WRKY transcription factors found in the network.

TABLE S4 Connectivity for Gene Regulatory Network. This dataset contains the degree/connectivity for each of the nodes within the network.

TABLE S5 DESeq2 output for stages 8 to 18. Differentially expressed genes found in *A. flavus* after using DESeq2.
